# Oxidative stress affects processing of amyloid precursor protein in vascular endothelial cells

**DOI:** 10.1371/journal.pone.0178127

**Published:** 2017-06-15

**Authors:** Abebe Muche, Thomas Arendt, Reinhard Schliebs

**Affiliations:** 1 Department of Human Anatomy, College of Medicine and Health Sciences, University of Gondar, Gondar, Ethiopia; 2 Paul Flechsig Institute for Brain Research, Medical Faculty, University of Leipzig, Leipzig, Germany; Massachusetts General Hospital, UNITED STATES

## Abstract

**Background:**

Oxidative stress is thought to be a key player in the pathogenesis of neurodegenerative dementia, including Alzheimer’s disease (AD). It has been assumed that oxidative stress contributes to the ß-amyloid deposition in cerebral blood vessels.

**Methods:**

In order to prove this hypothesis, we examined the effect of oxidative stress on the processing of amyloid precursor protein (APP) in primary endothelial cells (EC) derived from cerebral cortical tissue of transgenic Tg2576 mice. Following exposure of EC by 1 μM hydrogen peroxide for up to 48 hours, formation and secretion of APP cleavage products sAPPα and sAPPß into the culture medium as well as the expression of endothelial APP were assessed.

**Results:**

Oxidative stress resulted in enhanced secretion of sAPPß into the culture medium as compared to controls (absence of hydrogen peroxide), which was accompanied by an increased APP expression, induction of VEGF synthesis, nitric oxide and oxygen free radicals productions, and differential changes of endothelial phospo-p42/44 MAPK expression.

**Conclusion:**

The data suggest that oxidative stress may represent a major risk factor in causing Aß deposition in the brain vascular system by initiating the amyloidogenic route of endothelial APP processing. The enhanced β-secretase activity following oxidative stress exposure, possibly promoted by phosphorylation of p42/44 MAPK.

## Introduction

During the last decades a vast number of studies provided evidence that increased levels of oxidative stress occurs in conditions of neurodegeneration and even during brain aging (for reviews, see e.g., [1, 2]). In neurological disorders such as multiple sclerosis, stroke, neuroinfection as well as neurodegenerative diseases, including Alzheimer’s disease, oxidative stress is thought to be a principal player and an early event that trigger the progression of the pathogenic mechanisms.

However, the relatively non-specific nature of oxidative stress and observations that anti oxidant-based therapies appeared to be largely ineffective [3, 4], raised doubts whether oxidative stress is a cause or consequence of neurodegenerative events [2]. Particularly, it has been suggested that non-neuronal cells may also initiate and/or participate in oxidative stress-induced neurodegeneration. In this respect, the cerebrovasculature came into focus as one of the main source, but is also a major target of oxidative stress. The cerebral endothelial cells (EC) have unique structural and functional features and form together with surrounding astrocytes, neurons, pericytes, and vascular smooth muscle cells the neuronal-glial vascular unit. The neuronal-glial vascular unit is a complex anatomical and functional unit of the brain to maintain local metabolic homeostasis [5, 6]. Increased level of oxidative stress may lead to cerebrovascular changes that may serve as the initiator for oxidative stress-induced degeneration of associated neurons (for reviews, see e.g., [2, 7]).

Cerebrovascular abnormalities such as thickening of the microvascular basement membranes, decreased luminal diameter, and microvascular degeneration, as well as accumulation of β-amyloid (Aβ) in blood vessels (cerebral amyloid angiopathy) have frequently been observed in Alzheimer patients [7, 8]. Vascular Aβ deposits have been assumed to cause the degeneration of arterial vessels and cerebral capillaries, presumably mediated through the induction of reactive oxygen species (ROS) by activation of NADPH oxidase. Subsequently, the vascular Aβ deposition may impaire the blood brain barrier and severely affect regulation of cerebral blood vessels and brain perfusion [9–11].

The cerebral amyloid angiopathy is likely caused by the failure of Aβ elimination from the brain parenchyme [12], while a role of vascular smooth muscle and endothelial cells by abnormal processing of endothelial amyloid precursor protein (APP) has also been suggested [13, 14].

Thus, the question arises of what causes the abnormal processing of endothelial APP in Alzheimer’s disease. Based on recent studies that oxidative stress may enhance the expression and activity of beta-site APP cleavage enzyme-1 (BACE-1) in neurons [15–17], the present study stresses the hypothesis whether oxidative stress is also one of the players in initiating cerebral amyloid angiopathy by affecting endothelial APP metabolism. To address this hypothesis, following the exposure of primary cerebral endothelial cells (EC) derived from transgenic Tg2576 mouse brain to oxidative stress, the secretion of sAPPα and sAPPβ and expression of full length of APP was examined.

## Materials and methods

### Preparation of primary cerebral endothelial cell cultures

Primary cerebral microvascular endothelial cells (EC) were isolated from 2-month-old Tg2576 mouse brains (containing as transgene the human APP695 with the double mutation (K670N, M671L, Hsiao et al. [18], according to the method by Ichikawa et al. [19], which was already described previously [20].

The primary cerebral tissue of Tg2576 mouse brains was placed into 70% ethanol, moved into ice-cold PBS (-) and then rinsed five times in ice-cold Standard isolation medium (SIM: Ml99 containing 20 mM HEPES, 25 mM sodium bicarbonate, penicillin streptomycin mixture (50 I.U./ml, 50 μg/ml), amphotericin B (2.5 μg/ml), heparin (10 U/ml), and 5% FBS, and adjusted to a pH 7.4). The cerebral hemispheres were carefully dissected out and finely minced into small pieces, and then added with serum free SIM (SFSIM). The suspension was centrifuged at 1000 g, at 4°C for 10 minutes. The resulting pellet was resuspended in 0.02% dispase solution (in SFSIM) and incubated at 37°C for 60 minutes. The dispase suspension was again centrifuged at 1000 g, at 4°C for 10 minutes, and the pellet was resuspended in 15% dextran solution (in SIM). After centrifugation at 4500 g, 4°C for 10 minutes, the fat pad and dextran solution were collected into a new tube and centrifuged once again. The two pellets were collected and resuspended in the new dextran solution and centrifuged again. The resulting tissue pellet was resuspended in SFSIM and filtered through a 300 μm mesh. The microvessel suspension was dissociated using 0.1% collagenase / dispase (in SFSIM) at 37°C for 30 minutes. After the enzyme treatment, the microvessels were collected by centrifugation (1000 g, 4°C for 10 minutes), and resuspended in SIM. The suspension was layered onto a Percoll gradient formed by centrifugation of 50% isotonic Percoll at 25,000 x g, at 4°C for 70 minutes, and then centrifuged at 1650 x g, at 4°C for 10 minutes. After the Percoll gradient centrifugation, three layers could be observed. The endothelial cell fragments formed a band around the middle third of the gradient, and the entire middle layer was collected from the gradients. The pellet was washed three times in SIM. The preparation was resuspended in Growth Medium-A (GM-A: DMEM containing 20 mM sodium bicarbonate, 2 mM L-glutamine, ECGF (150 μg/ml), penicillin-streptomycin mixture (50 I.U./ml, 50μg/ml), amphotericin B (2.5 μg/ml), MC-210 (0.5 μg/ml), heparin (10 U/ml), and 20% FBS and adjusted to a pH 7.4.). The cell suspension was seeded onto collagen-coated 35-mm tissue culture dishes or 24-well tissue culture plates at a density of 50,000 cells/cm^2^. The cells were incubated in a moist 5% CO_2_, 95% air atmosphere, at 37°C. After the cell attachment, the cells were maintained according to the method of Gordon et al. [21] with minor modifications. For the first 3 days, the cells were cultured in GM-A and then cultured in GM-B (DMEM containing 20 mM sodium bicarbonate, 2 mM L-glutamine, penicillin-streptomycin mixture (50 I.U./ml, 50 μg/ml), amphotericin B (2.5 μg/ml), ECGF(150 μg/ml), 5% FBS, and 5% donor horse serum and adjusted to a pH 7.4.) after day 3. The culture medium was changed every other day until the cells became a confluent monolayer.

**Fig 1:**
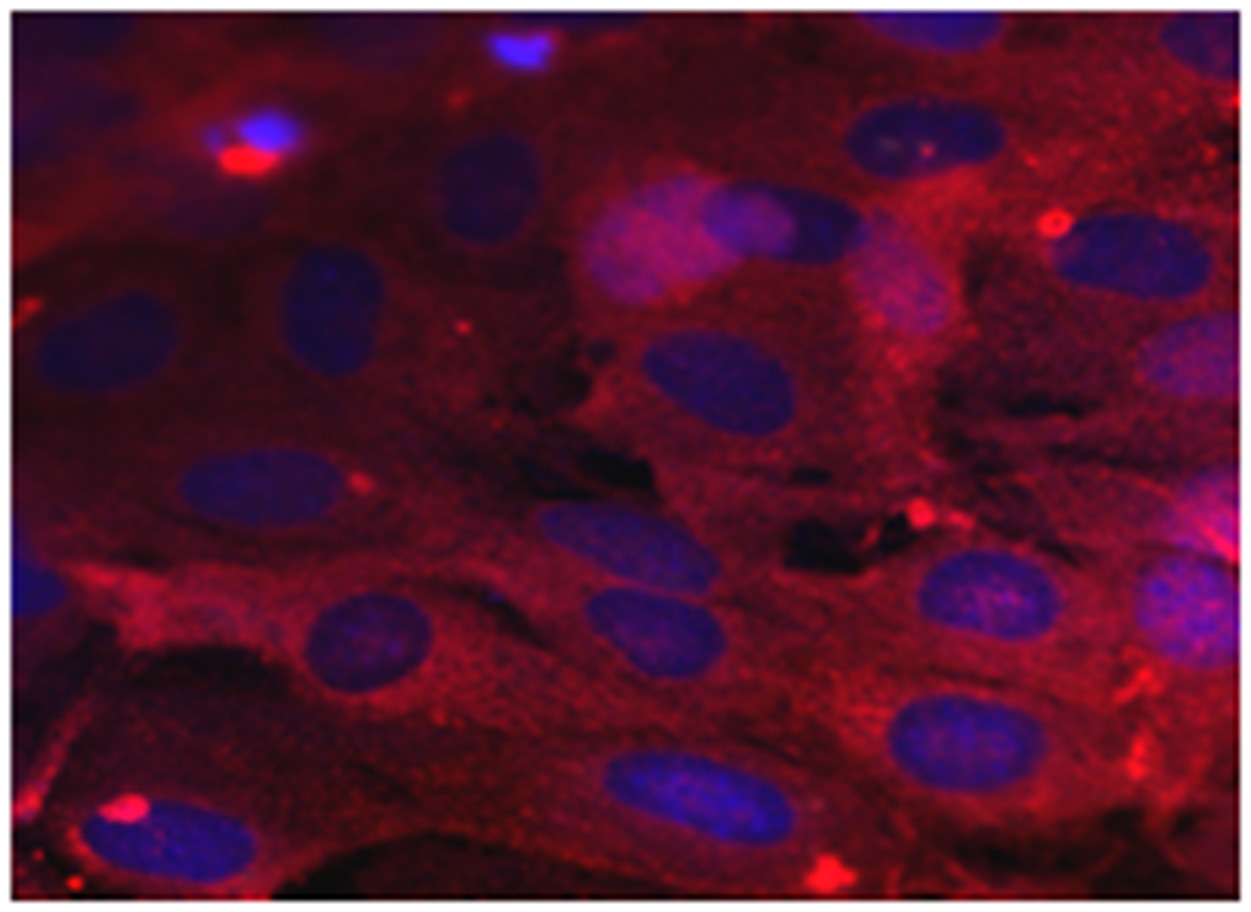
Representative example of EC immunostained with combination of primary antibodies directed against AWF (marker protein of EC, red fluorescence) and anti-GFAP (marker protein for astrocytes; green fluorescence), and counterstained with TOPRO-3 (blue cell nuclei). Note primary EC free of both primary astrocytes. Scale bar: 50 μm.

The purity of isolated EC was checked by subjecting sample preparations to immunocytochemistry using an antiserum directed against the Von-Willebrandt Factor (Factor VIII-related antigen, Sigma-Aldrich, Germany), a reliable marker for EC (Fig 1). Cross-checking revealed that the primary EC cultures used for the experiments did not contain significant amounts of neurons and/or astrocytes.

### Treatment of EC culture and tissue preparation

Oxidative stress was induced by incubating EC in the presence of varying concentration of hydrogen peroxide. In preliminary experiments EC were exposed by increasing concentrations of H_2_O_2_ ranging from 1 μM to 25 μM in order to check for an optimal concentration with lowest effect on cellular degeneration. Exposure of EC by 1 μM, 2.5 μM, 5 μM and 25 μM of H_2_O_2_ for up to 6 hours resulted in cell losses by about 10 ± 9%, 20 ± 7%, 35 ± 5%, and 40 ± 6%, respectively, as revealed by the MTT reduction assay (for experimental details, see [22], performed at the end of incubation. Based on these results for all further exposure experiments a concentration 1 μM H_2_O_2_ was chosen.

EC were incubated with 800 μl stimulation medium/ well in the presence of 1 μM of H_2_O_2_ for up to 48 hours as indicated. Control incubations were treated similarly but in the absence of any drug. Following incubations, the culture medium was carefully separated from the cell layer, centrifuged at 1000 x g for 10 minutes to remove cell debris. The supernatant was stored at -20°C pending quantitation of VEGF, sAPPβ and sAPPα present in the medium.

Cells attached at the bottom of the well, were properly scraped off, homogenized by sonification in 400 μl/well of PBS, divided in aliquots, and stored at -20°C. Aliquots of cell preparations were used for Western blotting, and to assay the protein content as described elsewhere [23].

### Cell viability assay

Cell viability was assayed by MTT-reduction. At the end of incubation period, the medium in each well was displaced by medium supplemented with 10% (V/V) 3-(4,5-dimethylthiazol-2-yl)-2,5-diphenyltetrazolium-bromid (MTT) stock solution (0.25% (w/v) in 0.1 M phosphate buffered saline, pH 7.4) and the incubation was continued for another 2 hours at 37°C. The medium was removed and 100 μl lysis solution (dimethyl sulfoxide/ethanol 1:1) was added to the cells. Following solubilisation, absorption at 562 nm was measured using a plate spectrophotometer. Determinations were performed in triplicates. Data are expressed as percentage change in absorption of control (= 100%, absorption detected in the absence of any drug in the incubation medium).

### Griess reaction for reactive nitrogen species (RNS) assay

Griess reaction was used to estimate the level of RNS released into the culture medium following exposure of EC by H_2_O_2_ [24].

In brief, 40 μl of different concentration of standards (25 μM; 12.5 μM; 6.25 μM; 3.125 μM; 1.562 μM and 0.781 μM) prepared from stock standard solution of 562.5 μM NaNO_2_ and samples were added to 860 μl of phosphate buffer (pH 7.4) containing 17 mM of sulfanil acid (SERVA) and 399.9 mM of naphthylenediamine dihydrochloride (Merck) in a cuvette. After three minutes room temperature incubation of the solutions, the absorbance was measured at wavelength of 496 nm. Then, the level of NO was calculated by Beer’s law (extinction coefficient of 660 M^-1^cm^-1^). Subsequently, ten microlitre of phosphoric acid (H_2_PO_4_) was added into each cuvette, mixed thoroughly and incubated for 15 minutes in the dark place at room temperature and the absorbance was quantified at 540 nm using a spectrophotometer. The level of NO_x_^-^ was calculated using a calibration curve of standards versus absorbance and expressed as μM.

### Thiobarbituric acid reactive substances (TBARS) assay

One hundred microliter of cell homogenate was placed into a labeled 1.5 mL microcentrifuge tube. Then, 200 μL ice cold 10% Trichloroacetic acid (TCA) was added to the 100 μL of each sample. The samples were incubated on the ice and centrifuged at 14,000 rpm in an Eppendorf centrifuge for five minutes each, respectively. Subsequently, 200 μL of each clear supernatant diluted in a factor of three was transferred into a newly labeled tube.

For colorimetric assay, the water bath was set to the temperature of 100°C. Then, 300 μl of different concentration of standards (30 μM; 18 μM; 9 μM; and 0 μM) were prepared from stock standard solution of 30 μM malondialdehyde (MDA). Out of the prepared solutions, only 200 μL standards and samples were transferred into separately labeled 1.5-mL screw cap tubes. To each of the standards and samples 200 μL of thiobarbituric acid (TBA) reagent was added and vortex and incubated at 100°C for 60 min. Then, the tubes were cooled to room temperature and briefly centrifuged. From each tube, 100 μL of solution was loaded in duplicate to wells of a clear bottom 96-well plate. The average absorbance for each standard samples was quantified at 540 nm using a plate spectrophotometer and the calibration curve plot was constructed. Finally, the TBARS concentration for each sample was calculated from the standard curve as follows: odds ratio of blank subtracted from odds ratio of sample divided by slope and multiplied by dilution factor.

### Assay of VEGF level

The secretion of VEGF into the medium of primary EC was examined using a commercially available ELISA kit on the basis of the manufacturer’s protocol (R&D Systems, Germany). The amount of VEGF was calculated by comparison with a standard curve, and expressed as pg/ml [22, 25].

### Assay of sAPPα and sAPPβ level

Following the experimental treatment, the amount of sAPPα and sAPPβ level, human APP cleavage products, released into the EC culture medium were determined using commercially available solid phase sandwich ELISAs (IBL Hamburg, Germany). It was performed according to the manufacturer’s protocol. Mathematically, the secretion of sAPPα and sAPPβ into the medium was determined in comparison with a standard curve of recombinant sAPPα, and sAPPβ, respectively, and expressed as ng/ml [22, 25].

The protein content of the particular cell homogenate measured at the end of incubation was used as a control to minimize the standard error of the data between wells and between experimental sessions.

### Western blot analysis to detect APP

Proteins of EC preparations were separated using standard SDS-polyacrylamide gel electrophoresis (PAGE; 10% polyacrylamide-gel), minigels (Miniprotean; Bio-Rad, Munich), subsequently, transferred to a nitrocellulose-membrane (Protean BA85, pore size 45 μm; Schleicher & Schüll) using a Bio-Rad tankblot system. Membranes were blocked for 60 minutes at room temperature with 4% bovine serum albumin (BSA) in Tris-buffered saline containing 0.1% Tween 20 (TBST) and incubated with the primary antibodies overnight at 4°C. The following primary antibodies were applied: the monoclonal antibodies anti-β-APP (22C11; Chemicon International, 1:2500), anti-mouse β-actin (Sigma, Munich, Germany, 1:10000), rabbit anti- Phospho- p42/44 MAPK or rabbit anti-Phospho- SAPK/JNK (Cell Signalling Technology, 1:1000) and polyclonal antibodies rabbit anti-p42/44 MAPK or rabbit anti-SAPK/JNK (Cell Signalling Technology, 1:1000). The blots were washed three times for 5 minutes with (TBST). The secondary antibody conjugated with horseradish peroxidase (goat-anti-mouse) was incubated for 60 minutes at room temperature, then washed three times with TBST. Protein bands were visualized by enhanced chemiluminescence detection (Western Blotting Luminol Reagent, Santa Cruz Biotechnology), according to the manufacturer’s protocol. Moreover, for the MAPK, the blots were stripped in stripping buffer for 30 minutes at 60°C and incubated with the antibody against actin, which is constitutively expressed in our cellular models.

Immunoblots were quantitatively evaluated by computer-assisted densitometry using the software package TINA (RAYTEST, Berlin). The optical density level of each protein band of interest was normalized to the corresponding expression level of β-actin. Normalized APP expression data are expressed as percentage of corresponding controls in the absence of any drug [22].

### Statistical analysis

All data were analyzed using SPSS version 20 statistical software. The difference between the experimental groups and corresponding controls were tested using one-way analysis of variance (ANOVA), followed by a Student’s t-test analysis using SPSS software. The data were presented as mean ± SEM. P-values < 0.05 were considered as statistically significant.

## Results

### Cell viability

To induce oxidative stress, EC were incubated in the presence of 1 μM of H_2_O_2_ for varying period of incubation times. Using the MTT reduction assay, the incubation time dependent consequences of 1 μM of H_2_O_2_ exposure on the cell viability was examined in parallel experiments at the end of incubations.

Exposure of EC to 1 μM of H_2_O_2_ resulted significant loss of viable cells by about 20% (P< 0.05) and 30% (P< 0.05) following incubation times of 24 hours and 48 hours, respectively, as compared to the corresponding control incubated in the absence of any drug.

### Oxidative stress affects expression and processing of APP as well as the ratio of sAPPβ and sAPPα

To reveal whether oxidative stress affects the metabolism of APP, EC were exposed by hydrogen peroxide. Following the completion of the incubation time, the cleavage products of APP such as sAPPß and sAPPα released into the culture medium were determined using the commercially available ELISA kits.

**Fig 2:**
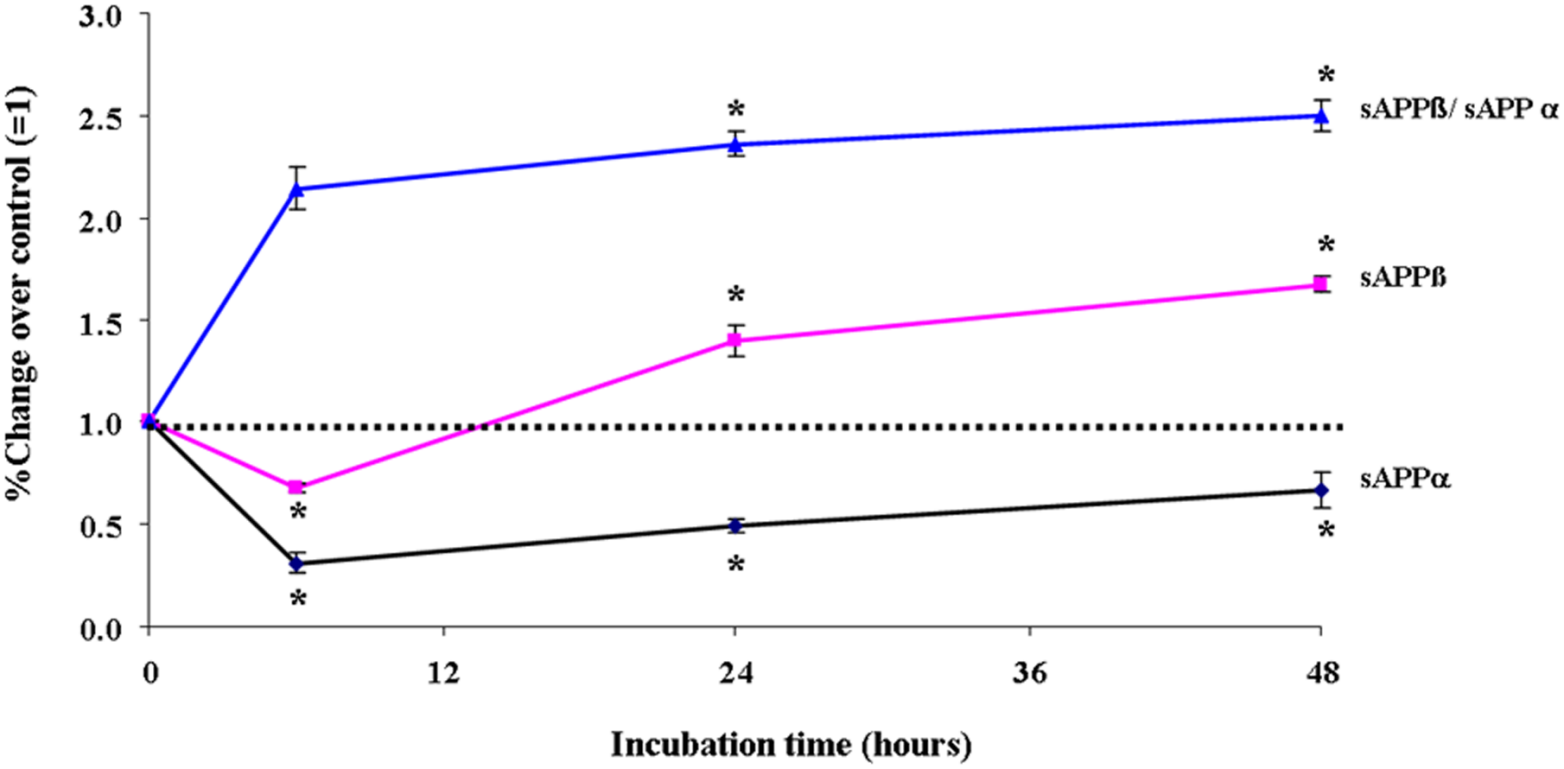
Effect of exposure of primary EC by 1 μM of H_2_O_2_ on release of sAPPß, and sAPPα into the culture medium. The ratio of corresponding amounts of sAPPß and sAPPα is plotted against the incubation time. Data are expressed as x-fold of control (= 1.0; incubations in the absence of any drug), and represent the mean ± SEM of six separate experiments per N = 6. *P<0.05 vs. control, two-tailed Student’s t-test.

Exposure of EC by 1 μM of H_2_O_2_ resulted in increased release of sAPPßswed into the culture medium by about 25% and 40% (P<0.05) after 24 and 48 hours incubations, respectively, when compared to control incubations in the absence of any drug (Fig 2). On the other hand, exposure of EC to 1 μM of H_2_O_2_ decreased the secretion of sAPPα into the culture medium by about 70%, already detectable 6 hours after incubation, as compared to the corresponding control. However, further incubation apparently diminished the inhibitory effect of H_2_O_2_ on sAPPα. Indeed, following 24 hours and 48 hours incubation, the secretion of sAPPα in the culture medium of 1 μM of H_2_O_2_ treated cells were by 50% and 30%, respectively, less than that detected in the corresponding control cultures (Fig 2). Subsequently, the oxidative stress shift the route of APP processing towards the amyloidogenic pathway, the ratio of the amount of sAPPßswed and sAPPα released into the culture medium is plotted versus corresponding incubation time (Fig 2). Indeed, oxidative stress tends to promote the amyloidogenic pathway of endothelial APP processing.

### Oxidative stress induces up-regulation of APP expression

**Fig 3:**
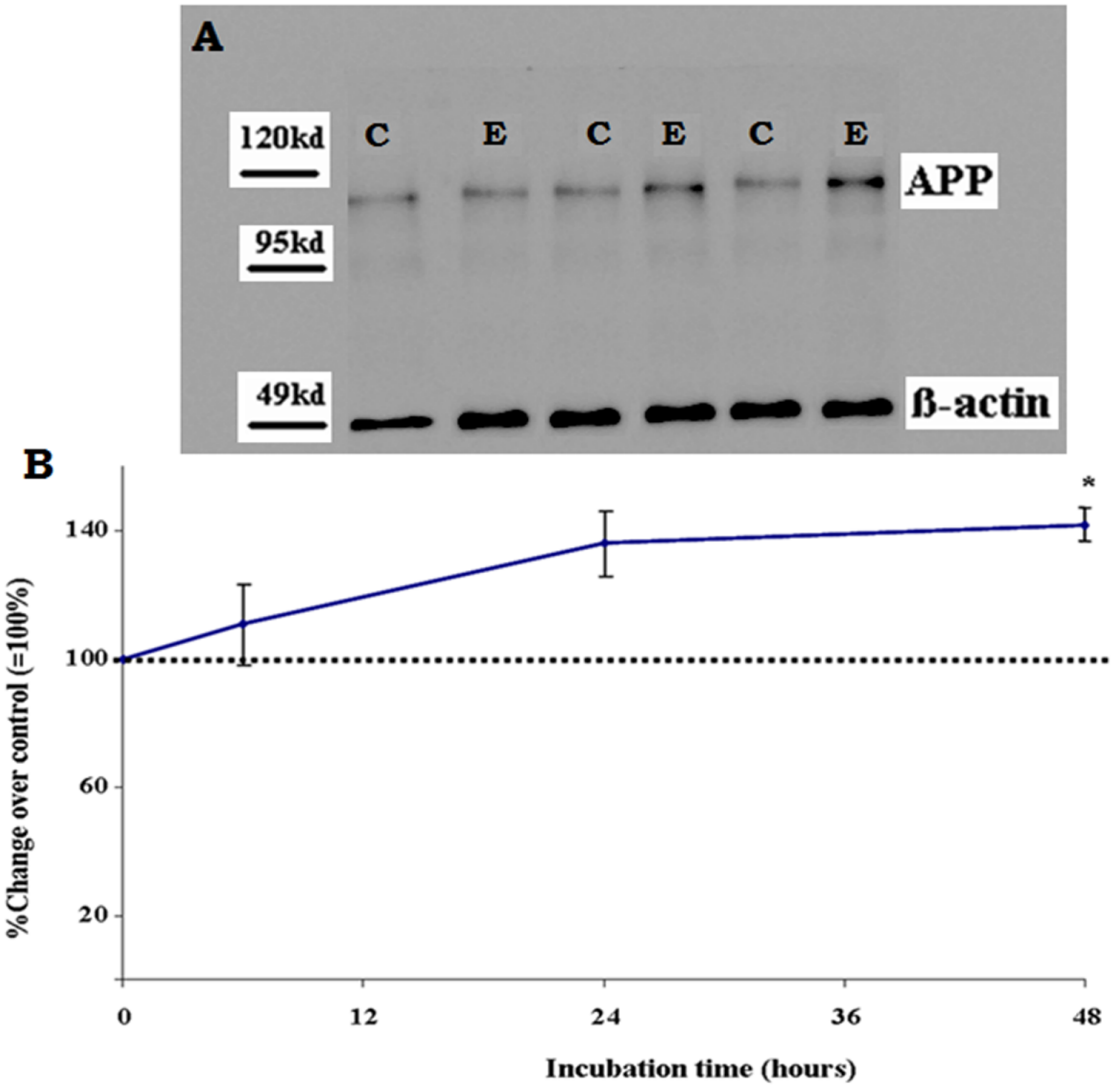
Effect of exposure of primary EC by 1 μM of H_2_O_2_ for up to 48 h on endothelial expression of amyloid precursor protein (APP). Results of densitometric analysis of immunoblots for APP (normalized against β-actin) are given as percentage change of corresponding controls incubated in the absence of H_2_O_2_ and represents the mean ± SEM of six separate experiments. *P < 0.05 vs. control, two-tailed Student’s t-test.

At the end of incubation time, the samples of cell homogenates of primary EC were subjected to semiquantitative Western analysis for APP. Densitometric evaluation of the blots demonstrated that oxidative stress gradually increased the level of APP expression with incubation time. Following 48h of incubation, the expression level of cellular APP in H_2_O_2_-exposed EC was significantly increased by 42 ± 6% (P<0.05) as compared to cells incubated under normal conditions in the absence of hydrogen peroxide (Fig 3).

### Oxidative stress induces up-regulation and secretion of both VEGF and RNS, and ROS

To reveal whether the primary EC used in this study are sensitive to oxidative stress, the production and release of both VEGF and RNS, including nitric oxide into the culture medium and ROS expression was examined using commercially available ELISA kit, Griess reaction and TBARS kit, respectively.

**Fig 4:**
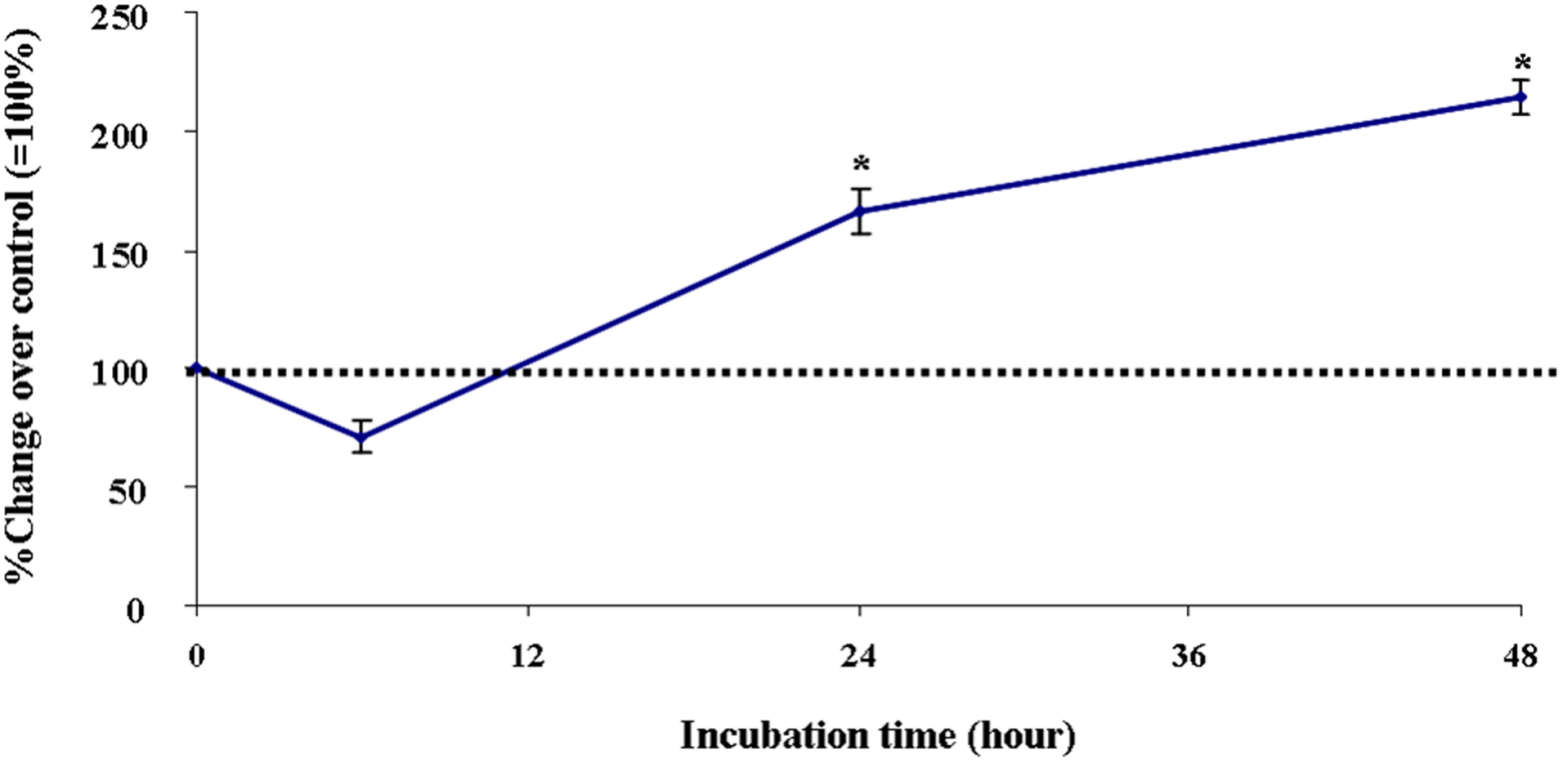
Effect of exposure of primary EC by 1 μM of H_2_O_2_ on release of endothelial VEGF into the culture medium with increasing incubation time. Data are expressed as percentage change of control (= 100%, incubations in the absence of any drug), and represent the mean + SEM of six separate experiments. *P<0.05 vs. control, two-tailed Student’s t-test.

Exposure of primary EC by 1 μM of H_2_O_2_ for 24 hours and 48 hours led to increased release of VEGF into the culture medium by about 60% and 110% (P<0.05), respectively (Fig 4).

**Fig 5:**
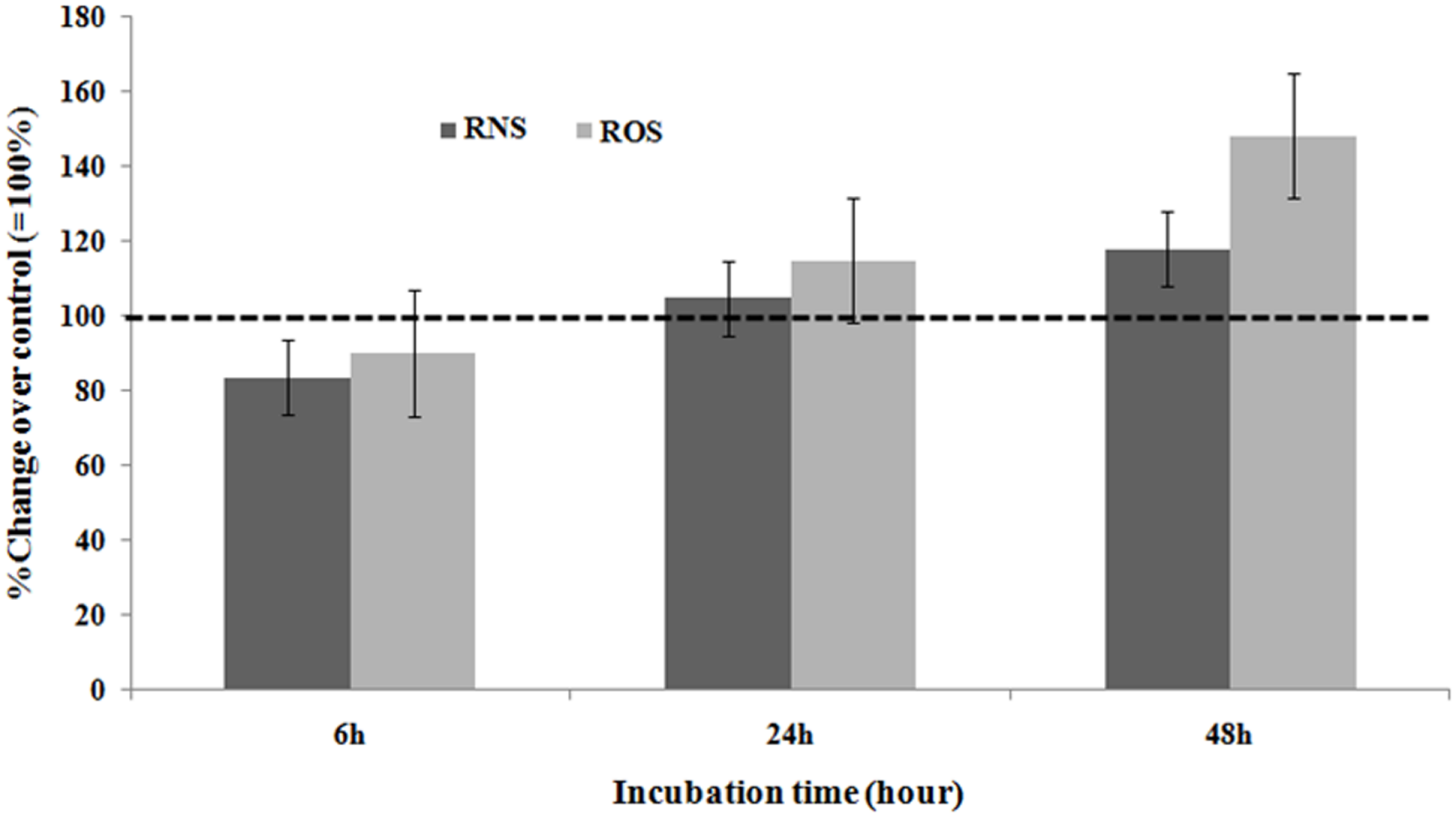
Effect of exposure of cultured primary EC by 1μM H_2_O_2_ with increasing incubation time on the release of RNS into the culture medium and production of ROS in the cell homogenate. Data are expressed as percentage change of control (= 100%; incubations in the absence of any drug), and represent the mean ± SEM of six separate experiments. *P<0.05 vs. control, one- way-ANOVA followed by Student’s t-test.

Stimulation of EC by 1 μM of H_2_O_2_ resulted in slightly increased generation and release of RNS by 18 ± 7% (P<0.05) but not earlier as after 48 hours of incubation (Fig 5).

Following the incubation of EC to 1 μM of H_2_O_2_ for 48 hours enhanced the production of ROS in the cell homogenate by 48 ± 9% (P<0.05) (Fig 5).

### Effect of oxidative stress on endothelial MAPK signaling

To disclose whether the changes in APP expression and processing induced by oxidative stress (as mimicked by H_2_O_2_ exposure), are interrelated with intracellular MAPK signaling cascades, cell homogenates of cultured EC were subjected to SDS-PAGE, blotted, and immunostained for key enzymes of MAPK signaling cascades (p42/44 and JNK), including their phosphorylated forms.

The densitometric evaluation of the immunoblots revealed that the exposure of EC cultures by H_2_O_2_ affected neither expression of phosphorylated nor non-phosphorylated forms of JNK as compared to the corresponding controls, regardless of the incubation time examined (not shown).

**Fig 6:**
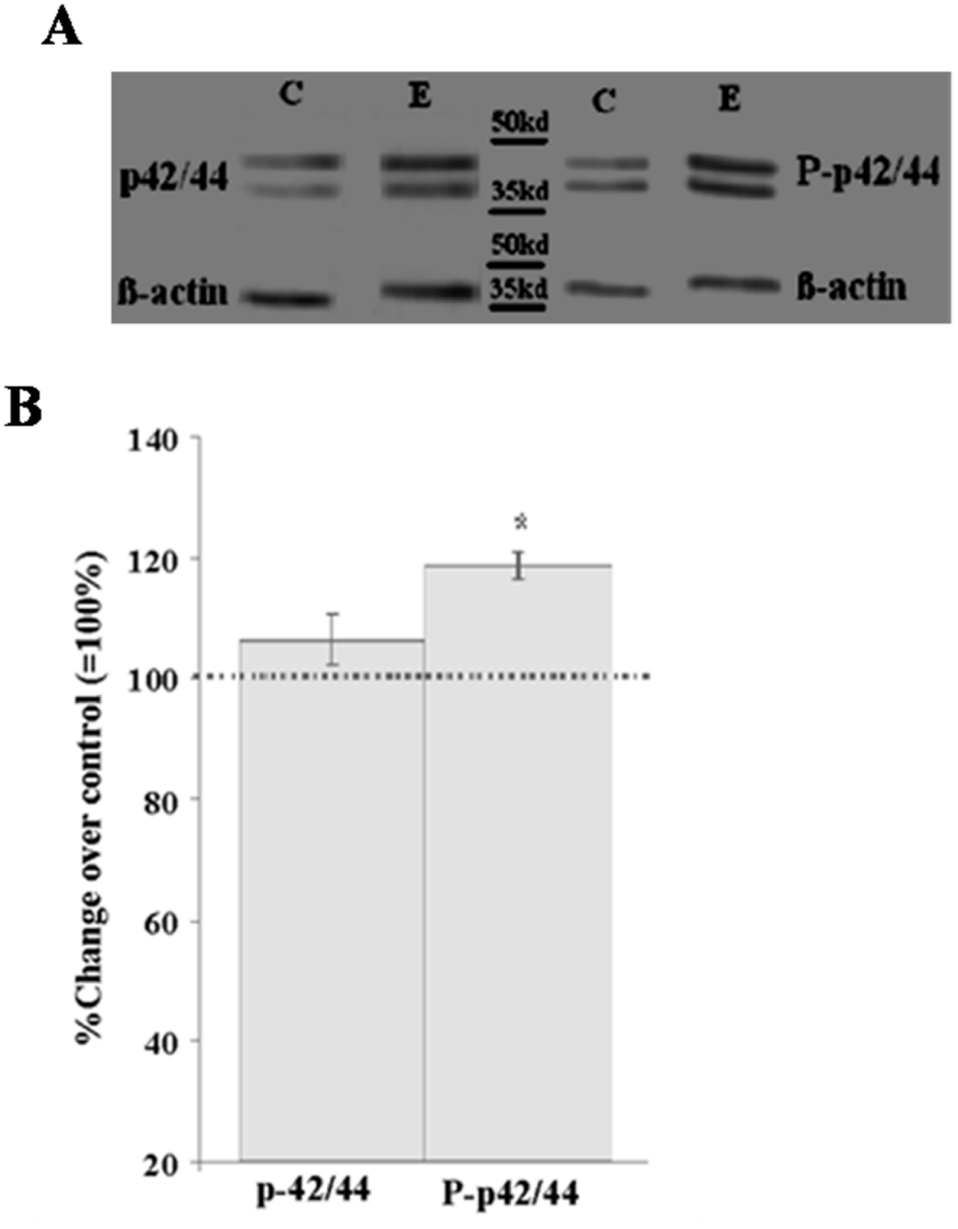
Effect of exposure of primary EC by 1 μM of H_2_O_2_ for up to 48h on endothelial expression of p42/44 and phophorylated p42/44 (P-p42/44) MAPK. Results of densitometric analysis of immunoblots for p42/44 and phospho-p42/44 MAPK (normalized against β-actin) are given as percentage change of corresponding controls incubated in the absence of H_2_O_2_ and represents the mean ± SEM of six separate experiments. *P < 0.05 vs. control, two-tailed Student’s t-test.

In contrast, the expressions of p42/44 and Phospho-p42/44 MAPK were found to be differentially affected when EC were subjected to H_2_O_2_ exposure as compared to the corresponding control incubations (Fig 6).

The Western blot analysis demonstrated that the exposure of primary EC by 1 μM of H_2_O_2_ for up to 24 hours did not affect the expression level of Phospho-p42/44 MAPK, while further exposure for up to 48 hours resulted in an up-regulation of Phospho-p42/44 MAPK by about 30% (P<0.05), compared to the expression in control incubations in the absence of any drug. In contrast, incubation of EC with 1 μM of H_2_O_2_ did not affect the expression level of p42/44 MAPK as compared to the corresponding control incubation in the absence of H_2_O_2_ (Fig 6).

## Discussion

The present study was undertaken to disclose whether oxidative stress may contribute to the cerebral amyloid angiopathy [8, 14] by affecting endothelial APP processing. In order to prove our hypothesis primary cerebral ECs derived from transgenic Tg2576 mouse brain, were subjected to oxidative stress mimicked by 1 μM H_2_O_2_, followed by assessment of the formation and secretion of APP cleavage products such as sAPPα and sAPPβ. The main finding of this study is that oxidative stress alters the ratio of α-, and β-secretory processing of endothelial APP by favoring the amyloidogenic route of APP metabolism, which is accompanied by concomitantly increased secretion of VEGF, generation of NO and ROS, and changes in Phospho-p42/44 MAPK expression.

However, exposure of EC by 1 μM H_2_O_2_ is also accompanied by a slight loss of cells during incubation which may result into falsification when comparing data obtained in control and experimental sessions. Reductions of a certain parameter assayed following hydrogen peroxide exposure may solely be due to the lower number of living cells in the wells compared to control incubations, while increases may represent an underestimation of the real value. As we found an oxidative stress gradually increased the level of APP expression with incubation time. Besides, it increases the release of sAPPβ and the expression of Phospho-p42/44 MAPK following H2O2 exposure, the real enhancements of these parameters may even be higher.

The primary EC used in this study were obtained from brain tissue of 2-month-old transgenic Tg2576 mice. The Tg2576 mouse brain demonstrates about a six-fold higher expression of the human APP transgene as compared to the endogenous murine APP [18], which makes assessments of APP cleavage products more accessible, and allows to exploit the greater availability of antibodies directed against human APP cleavage products.

While there are a number of studies demonstrating that oxidative stress participates in events, enhancing amyloidogenic APP processing in neurons [17, 26], the present study describes for the first time a role of oxidative stress in affecting cerebrovascular endothelial APP processing. As ECs play an essential role in forming the blood brain-barrier, they also exhibit a major target for oxidative stress stimuli. Thus, the findings provide evidence of a partly endogenous source of Aβ deposits in the CAA by stress-induced changes in endothelial APP metabolism.

In spite of uncertain molecular mechanism yet, the APP pathway enzymes, α-, and β-secretases are widely expressed during transcription, translation, and post-translation [27–30]. However, signal molecules such as RNS,VEGF or ROS may play a role in affecting those signaling pathways that mediate secretase activity beyond transcriptional and translational control, e.g. G-protein coupled receptor-, MAPK-, and tyrosine kinase signaling.

The oxidative stress-induced VEGF secretion was in coincidence with increased secretion of sAPPß, and decreased level of sAPPα, suggesting a link between VEGF and APP processing. Interestingly, studies done on brain slice culture, as well as on primary neuronal cell cultures derived from Tg2576 mice supported the association between VEGF and APP processing [22, 25].

Prolonged exposure of primary EC by 1 μM of H_2_O_2_ for up to 48 hours resulted in the up-regulation of NO and ROS suggesting also a link with vascular endothelial APP processing, which is supported by a recent study demonstrating that regulation and activity of BACE1 is potentially modified by NO via transcriptional and posttranscriptional mechanisms [31]. Similarly, a microdialysis study in experimental rats subjected to acute ischemia demonstrated a direct involvement of NO in up-regulation of hippocampal BACE1 expression and Aß production [32]. The developmental temporal coincidence of increased levels of NO and RNS with the onset of Aβ plaque deposition in Tg2576 mice further suggests the role of NO to trigger β-secretase expression and activity [33]. Furthermore, Oxidative stress which is common denominator for AD enhances up-regulation BACE1 and activate the enzyme, resulting in excessive cleavage of APP and Aβ generation [34–36].

MAPK pathways play a pivotal role in processing and secretion of APP [37–39]. In the present study, exposure of EC by 1 μM H_2_O_2_ for 48 hours resulted into the enhanced expression of phospho-p42/44 MAPK suggesting an oxidative stress-mediated phosphorylation of the p42/44 MAPK. It implicates a potential role of MAPK signaling on the regulation of activities of secretases. Indeed, several reports indicate the involvement of MAPK pathway in APP metabolism, including activity and expression of secretases. A recent study in PC12 cells reported that the insulin-like growth factor-induced reduction of BACE-1 is presumably mediated through the MAPK/ERK1/2 signaling pathway [40]. In NSE/hAPP-C105 transgenic mice treated with green tea catechin a down-regulation of γ-secretase activity and of Aß42 mediated via MAPK signaling has been observed [41]. In addition, studies in transgenic rat expressing human selenoprotein, treatment of selenium induced the activity of α/γ secretase via activation of the ERK pathway [42].

Regardless of the incubation times of the present study, the expression of either phosphorylated or non-phosphorylated forms of JNK in EC were not influenced by the exposure EC cultures to 1 μM of H_2_O_2_ compared to the corresponding controls. Tamagno and collaborators (2005), however, reported the up-regulation of BACE-1 by 4-hydroxynonenal (oxidative stress mediator) through the activation of JNK and p38-MAPK signaling [43]. This may indicate that the expression and activity of β-secretase regulated under the participation of various molecular mechanisms.

## Conclusion

In conclusion, the finding from the present study suggest that oxidative stress promotes the amyloidogenic pathway of endothelial APP processing driven by increasing the ratio of sAPPβ and sAPPα which subsequently enhance the accumulation of cerebrovascular amyloid protein. Probably, phosphorylation of p42/44 MAPK promotes amyloidogenic pathway following oxidative stress. However, the underlining mechanisms still require further investigation.
